# *N*,*N*′-Bis(1-ethynylcyclo­hexyl)­pyro­mellitic diimide

**DOI:** 10.1107/S1600536809030979

**Published:** 2009-08-08

**Authors:** Chenaimwoyo A. Gondo, Daniel E. Lynch, Darren G. Hamilton

**Affiliations:** a Department of Chemistry, Mount Holyoke College, South Hadley, Masssachusetts 01075, USA; b Exilica Limited, The Technocentre, Puma Way, Coventry CV1 2TT, England

## Abstract

The title compound, C_26_H_24_N_2_O_4_, consists of a symmetrical mol­ecule that lies across a crystallographic inversion centre. The C—C distance in the triple bond is 1.188 (2) Å and there is also an inter­molecular C—H⋯O contact from a terminal acetyl­ene C—H to one of the dimiide O atoms [3.4349 (19) Å].

## Related literature

For literature relating to the oxidative coupling of terminal acetyl­enes, see: Anderson, Anderson & Sanders (1995 ▶); Anderson, Walter *et al.* (1995 ▶); Hamilton *et al.* (1998 ▶); Raehm *et al.* (2002 ▶).
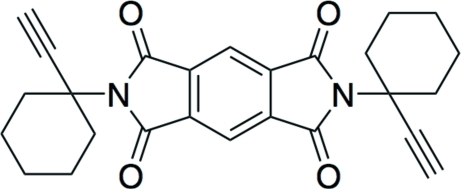


## Experimental

### 

#### Crystal data

C_26_H_24_N_2_O_4_
*M* *_r_* = 428.47
Monoclinic, 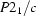

*a* = 13.1774 (3) Å
*b* = 7.1519 (1) Å
*c* = 11.8104 (3) Å
β = 112.495 (1)°
*V* = 1028.36 (4) Å^3^
*Z* = 2
Mo *K*α radiation
μ = 0.09 mm^−1^
*T* = 120 K
0.40 × 0.35 × 0.20 mm

#### Data collection

Bruker–Nonius KappaCCD diffractometer
Absorption correction: multi-scan (*SADABS*; Sheldrick, 2003 ▶) *T*_min_ = 0.963, *T*_max_ = 0.982
12939 measured reflections
2022 independent reflections
1898 reflections with *I* > 2σ(*I*)
*R*_int_ = 0.033

#### Refinement

*R*[*F* ^2^ > 2σ(*F* ^2^)] = 0.065
*wR*(*F* ^2^) = 0.158
*S* = 1.29
2022 reflections
146 parameters
H-atom parameters constrained
Δρ_max_ = 0.60 e Å^−3^
Δρ_min_ = −0.80 e Å^−3^

### 

Data collection: *COLLECT* (Hooft, 1998 ▶); cell refinement: *DENZO* (Otwinowski & Minor, 1997 ▶) and *COLLECT*; data reduction: *DENZO* and *COLLECT*; program(s) used to solve structure: *SHELXS97* (Sheldrick, 2008 ▶); program(s) used to refine structure: *SHELXL97* (Sheldrick, 2008 ▶); molecular graphics: *PLATON97* (Spek, 2009 ▶); software used to prepare material for publication: *SHELXL97*.

## Supplementary Material

Crystal structure: contains datablocks I, global. DOI: 10.1107/S1600536809030979/zs2003sup1.cif

Structure factors: contains datablocks I. DOI: 10.1107/S1600536809030979/zs2003Isup2.hkl

Additional supplementary materials:  crystallographic information; 3D view; checkCIF report

## Figures and Tables

**Table 1:** Hydrogen-bond geometry (Å, °)

*D*—H⋯*A*	*D*—H	H⋯*A*	*D*⋯*A*	*D*—H⋯*A*
C14—H12⋯O2^i^	0.95	2.52	3.4349 (19)	161

Symmetry code: (i) 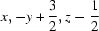.

**Fig. 1.**
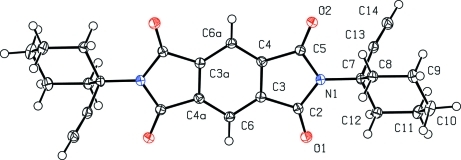
Molecular configuration and atom-numbering scheme for (I). Displacement ellipsoids are drawn at the 50% probability level. Symmetry code (a): -x, -y + 1, -z + 1.

## supplementary crystallographic information

### Comment

The oxidative coupling of terminal acetylenes has proven to be a valuable architectural tool in the preparation of large macrocycles, especially when coupled to a templating mechanism to organize the premacrocycle components (Anderson, Anderson & Sanders, 1995). In this manner some remarkable structures have been assembled with admirable efficiency, given the entropic handicap imposed on the synthesis of large ring macrocycles (Anderson, Walter *et al.*, 1995). One area in which a template greatly favors cyclization, and subsequently forms an integral part of the product structure, is in the synthesis of interlocked molecular compounds (catenanes and rotaxanes, Hamilton *et al.*, 1998). Numerous systems have been reported that rely on the attractive interaction between π-electron deficient aromatic diimides and π-electron rich aromatic diethers to establish the desired templating effect (Raehm *et al.*, 2002). In many of these instances the diimide component was equipped with terminal acetylenes, susbsequent oxidative coupling of which afforded the desired interlocked molecular systems. The title compound was prepared to address a key shortcoming of many acetylenic diimides of this type, namely their relatively low solubility in most organic solvents, in particular those in which the templating effects, so crucial to macrocycle synthesis, would be most effectively deployed. The presence of cyclohexyl substituents at the junctures of the diimide core with the acetylene substituents engendered high organic solvent solubility while retaining the key structural features required of the diimide unit. Reported here is the structure of the title compound (I), which is a symmetrical molecule that lies across a crystallographic inversion centre (Fig. 1), the asymmetric unit comprising half of the molecule. With such a simple molecule there are very few distinct features to report although it is worth mentioning that the C—C distance in the triple bond is 1.188 (2) Å. There is also an intermolecular C—H···O contact between the terminal acetylene C—H and one of the dimiide O atoms (Table 1).

### Experimental

To a stirred solution of 1,2,4,5-benzenetetracarboxylic dianhydride (2.18 g, 10 mmol) in dry THF (20 mL) was added a solution of 1-ethynylcyclohexylamine (2.50 g, 2.74 ml, 20 mmol) in dry THF (10 ml). After 6 h the reaction was evaporated to give a white foam to which was added acetic anhydride (30 ml). After heating at 130° C for 2 h the reaction was cooled to room temperature and poured into vigorously stirred icecold water. The precipitated solids were collected at the pump, washed with cold water, and recrystallized from aqueous DMF to afford pale yellow crystals of the title compound (0.71 g, 17%): m.p. 219–220° C; ^13^C NMR (100 MHz, CDCl_3_) δ 166.5, 138.0, 119.0, 83.0, 75.0, 60.0, 36.0, 25.0, 23.0; ^1^H NMR (400 MHz, CDCl_3_) δ 8.21 (s, 2H), 2.65 (s, 2H), 2.56–2.45, 2.44–2.29, 1.90–1.60, 1.40–1.20 (4 *x* multiplet, 20H). Single crystals of suitable quality for structure determination were grown by vapor diffusion of water into a DMF solution of the title compound.

### Refinement

All H atoms were included in the refinement at calculated positions, in the riding-model approximation, with C—H distances of 0.95 (CH) and 0.99Å (CH_2_). The isotropic displacement parameters for all H atoms were set equal to 1.25*U*_eq_ of the carrier atom. The large maximum and minimum residual electron density peaks [0.60 eÅ^-3^, 1.45 Å from C13 and -0.80 eÅ^-3^, 1.30 Å from H1 respectively] are unexplained.

### Crystal data

**Table:**

C_26_H_24_N_2_O_4_	*F*(000) = 452
*M**_r_* = 428.47	*D*_x_ = 1.384 Mg m^−^^3^
Monoclinic, *P*2_1_/*c*	Melting point = 492–493 K
Hall symbol: -P 2ybc	Mo *K*α radiation, λ = 0.71073 Å
*a* = 13.1774 (3) Å	Cell parameters from 2528 reflections
*b* = 7.1519 (1) Å	θ = 2.9–27.5°
*c* = 11.8104 (3) Å	µ = 0.09 mm^−^^1^
β = 112.495 (1)°	*T* = 120 K
*V* = 1028.36 (4) Å^3^	Prism, colourless
*Z* = 2	0.40 × 0.35 × 0.20 mm

### Data collection

**Table:**

Bruker–Nonius KappaCCD diffractometer	2022 independent reflections
Radiation source: Bruker Nonius FR591 rotating anode	1898 reflections with *I* > 2σ(*I*)
10 cm confocal mirrors	*R*_int_ = 0.033
Detector resolution: 9.091 pixels mm^-1^	θ_max_ = 26.0°, θ_min_ = 3.3°
φ & ω scans	*h* = −16→16
Absorption correction: multi-scan (*SADABS*; Sheldrick, 2003)	*k* = −8→8
*T*_min_ = 0.963, *T*_max_ = 0.982	*l* = −13→14
12939 measured reflections	

### Refinement

**Table:**

Refinement on *F*^2^	Secondary atom site location: difference Fourier map
Least-squares matrix: full	Hydrogen site location: inferred from neighbouring sites
*R*[*F*^2^ > 2σ(*F*^2^)] = 0.065	H-atom parameters constrained
*wR*(*F*^2^) = 0.158	*w* = 1/[σ^2^(*F*_o_^2^) + (0.091*P*)^2^ + 0.2895*P*] where *P* = (*F*_o_^2^ + 2*F*_c_^2^)/3
*S* = 1.29	(Δ/σ)_max_ = 0.001
2022 reflections	Δρ_max_ = 0.60 e Å^−^^3^
146 parameters	Δρ_min_ = −0.80 e Å^−^^3^
0 restraints	Extinction correction: *SHELXL97*, Fc^*^=kFc[1+0.001xFc^2^λ^3^/sin(2θ)]^-1/4^
Primary atom site location: structure-invariant direct methods	Extinction coefficient: 0.38 (3)

### Special details

**Table:**

Experimental. The minimum and maximum absorption values stated above are those calculated in *SHELXL97* from the given crystal dimensions. The ratio of minimum to maximum apparent transmission was determined experimentally as 0.798007.

### Fractional atomic coordinates and isotropic or equivalent isotropic displacement parameters (Å^2^)

**Table:**

	*x*	*y*	*z*	*U*_iso_*/*U*_eq_	
O1	0.06501 (9)	0.08401 (16)	0.34794 (11)	0.0222 (4)	
O2	0.22629 (9)	0.66713 (15)	0.40367 (11)	0.0202 (4)	
N1	0.16857 (10)	0.35785 (17)	0.36123 (11)	0.0156 (4)	
C2	0.08812 (12)	0.2440 (2)	0.37881 (13)	0.0158 (4)	
C3	0.03482 (12)	0.3636 (2)	0.44392 (13)	0.0151 (4)	
C4	0.07781 (12)	0.5432 (2)	0.45479 (13)	0.0152 (4)	
C5	0.16567 (12)	0.5404 (2)	0.40426 (13)	0.0158 (4)	
C6	−0.04412 (12)	0.3126 (2)	0.48923 (13)	0.0162 (4)	
H1	−0.0726	0.1893	0.4825	0.020*	
C7	0.25590 (11)	0.3002 (2)	0.31631 (13)	0.0150 (4)	
C8	0.36865 (12)	0.3129 (2)	0.42496 (14)	0.0169 (4)	
H2	0.3812	0.4431	0.4557	0.021*	
H3	0.3680	0.2316	0.4925	0.021*	
C9	0.46204 (12)	0.2530 (2)	0.38642 (15)	0.0208 (4)	
H4	0.4676	0.3424	0.3251	0.026*	
H5	0.5323	0.2563	0.4586	0.026*	
C10	0.44355 (13)	0.0570 (2)	0.33209 (16)	0.0250 (4)	
H6	0.4485	−0.0345	0.3969	0.031*	
H7	0.5020	0.0272	0.3017	0.031*	
C11	0.33156 (13)	0.0392 (2)	0.22690 (15)	0.0210 (4)	
H8	0.3201	−0.0920	0.1979	0.026*	
H9	0.3298	0.1191	0.1578	0.026*	
C12	0.23911 (12)	0.0975 (2)	0.26781 (13)	0.0169 (4)	
H10	0.2376	0.0120	0.3331	0.021*	
H11	0.1677	0.0875	0.1977	0.021*	
C13	0.25281 (12)	0.4268 (2)	0.21565 (14)	0.0178 (4)	
C14	0.25817 (13)	0.5152 (2)	0.13290 (15)	0.0211 (4)	
H12	0.2625	0.5858	0.0668	0.026*	

### Atomic displacement parameters (Å^2^)

**Table:**

	*U*^11^	*U*^22^	*U*^33^	*U*^12^	*U*^13^	*U*^23^
O1	0.0244 (6)	0.0177 (6)	0.0284 (7)	−0.0040 (4)	0.0144 (5)	−0.0057 (5)
O2	0.0223 (6)	0.0163 (6)	0.0262 (6)	−0.0021 (4)	0.0141 (5)	−0.0008 (4)
N1	0.0160 (6)	0.0157 (7)	0.0173 (7)	0.0003 (5)	0.0089 (5)	−0.0002 (5)
C2	0.0153 (7)	0.0177 (8)	0.0150 (7)	0.0000 (5)	0.0065 (6)	0.0006 (6)
C3	0.0151 (7)	0.0160 (8)	0.0138 (7)	0.0004 (5)	0.0051 (6)	0.0002 (5)
C4	0.0144 (7)	0.0165 (8)	0.0144 (7)	0.0004 (5)	0.0053 (6)	0.0019 (5)
C5	0.0167 (7)	0.0158 (8)	0.0153 (8)	0.0011 (5)	0.0068 (6)	0.0014 (5)
C6	0.0164 (7)	0.0147 (7)	0.0173 (8)	−0.0008 (5)	0.0064 (6)	−0.0003 (6)
C7	0.0150 (7)	0.0165 (8)	0.0159 (7)	0.0017 (5)	0.0085 (6)	0.0004 (6)
C8	0.0175 (8)	0.0182 (8)	0.0154 (8)	0.0004 (5)	0.0069 (6)	−0.0006 (6)
C9	0.0161 (8)	0.0257 (9)	0.0211 (8)	0.0010 (6)	0.0078 (6)	0.0001 (6)
C10	0.0205 (8)	0.0294 (9)	0.0251 (9)	0.0066 (6)	0.0086 (7)	−0.0039 (7)
C11	0.0227 (8)	0.0228 (9)	0.0185 (8)	0.0032 (6)	0.0091 (6)	−0.0038 (6)
C12	0.0181 (8)	0.0174 (8)	0.0159 (8)	−0.0001 (6)	0.0071 (6)	−0.0010 (6)
C13	0.0159 (7)	0.0189 (8)	0.0196 (8)	0.0027 (5)	0.0081 (6)	0.0001 (6)
C14	0.0219 (8)	0.0224 (8)	0.0217 (8)	0.0040 (6)	0.0111 (6)	0.0048 (7)

### Geometric parameters (Å, °)

**Table:**

O1—C2	1.2042 (19)	C8—H2	0.99
O2—C5	1.2100 (18)	C8—H3	0.99
N1—C5	1.4066 (19)	C9—C10	1.522 (2)
N1—C2	1.4136 (19)	C9—H4	0.99
N1—C7	1.4976 (18)	C9—H5	0.99
C2—C3	1.493 (2)	C10—C11	1.528 (2)
C3—C6	1.388 (2)	C10—H6	0.99
C3—C4	1.389 (2)	C10—H7	0.99
C4—C6^i^	1.387 (2)	C11—C12	1.530 (2)
C4—C5	1.492 (2)	C11—H8	0.99
C6—C4^i^	1.387 (2)	C11—H9	0.99
C6—H1	0.95	C12—H10	0.99
C7—C13	1.482 (2)	C12—H11	0.99
C7—C12	1.543 (2)	C13—C14	1.188 (2)
C7—C8	1.550 (2)	C14—H12	0.95
C8—C9	1.528 (2)		
			
C5—N1—C2	110.85 (12)	C7—C8—H3	109.4
C5—N1—C7	120.94 (12)	H2—C8—H3	108.0
C2—N1—C7	127.92 (12)	C10—C9—C8	111.50 (13)
O1—C2—N1	128.31 (14)	C10—C9—H4	109.3
O1—C2—C3	125.88 (13)	C8—C9—H4	109.3
N1—C2—C3	105.81 (12)	C10—C9—H5	109.3
C6—C3—C4	123.13 (14)	C8—C9—H5	109.3
C6—C3—C2	128.11 (14)	H4—C9—H5	108.0
C4—C3—C2	108.76 (13)	C9—C10—C11	111.64 (13)
C3—C4—C6^i^	122.54 (14)	C9—C10—H6	109.3
C3—C4—C5	107.58 (13)	C11—C10—H6	109.3
C6^i^—C4—C5	129.77 (14)	C9—C10—H7	109.3
O2—C5—N1	125.73 (14)	C11—C10—H7	109.3
O2—C5—C4	127.39 (14)	H6—C10—H7	108.0
N1—C5—C4	106.83 (12)	C10—C11—C12	111.00 (12)
C3—C6—C4^i^	114.32 (14)	C10—C11—H8	109.4
C3—C6—H1	122.8	C12—C11—H8	109.4
C4^i^—C6—H1	122.8	C10—C11—H9	109.4
C13—C7—N1	109.08 (12)	C12—C11—H9	109.4
C13—C7—C12	108.67 (12)	H8—C11—H9	108.0
N1—C7—C12	111.69 (12)	C11—C12—C7	110.79 (12)
C13—C7—C8	110.59 (12)	C11—C12—H10	109.5
N1—C7—C8	108.28 (11)	C7—C12—H10	109.5
C12—C7—C8	108.54 (12)	C11—C12—H11	109.5
C9—C8—C7	111.30 (12)	C7—C12—H11	109.5
C9—C8—H2	109.4	H10—C12—H11	108.1
C7—C8—H2	109.4	C14—C13—C7	172.85 (16)
C9—C8—H3	109.4	C13—C14—H12	180.0
			
C5—N1—C2—O1	176.47 (15)	C6^i^—C4—C5—N1	178.17 (14)
C7—N1—C2—O1	−9.7 (2)	C4—C3—C6—C4^i^	0.8 (2)
C5—N1—C2—C3	−3.03 (16)	C2—C3—C6—C4^i^	−179.81 (14)
C7—N1—C2—C3	170.78 (13)	C5—N1—C7—C13	−57.01 (17)
O1—C2—C3—C6	5.3 (3)	C2—N1—C7—C13	129.74 (15)
N1—C2—C3—C6	−175.23 (14)	C5—N1—C7—C12	−177.14 (12)
O1—C2—C3—C4	−175.27 (14)	C2—N1—C7—C12	9.6 (2)
N1—C2—C3—C4	4.25 (16)	C5—N1—C7—C8	63.40 (17)
C6—C3—C4—C6^i^	−0.8 (3)	C2—N1—C7—C8	−109.86 (16)
C2—C3—C4—C6^i^	179.65 (13)	C13—C7—C8—C9	−61.41 (16)
C6—C3—C4—C5	175.74 (13)	N1—C7—C8—C9	179.13 (12)
C2—C3—C4—C5	−3.77 (16)	C12—C7—C8—C9	57.70 (16)
C2—N1—C5—O2	178.38 (14)	C7—C8—C9—C10	−56.05 (17)
C7—N1—C5—O2	4.1 (2)	C8—C9—C10—C11	54.10 (18)
C2—N1—C5—C4	0.82 (16)	C9—C10—C11—C12	−54.98 (18)
C7—N1—C5—C4	−173.49 (12)	C10—C11—C12—C7	57.87 (17)
C3—C4—C5—O2	−175.58 (15)	C13—C7—C12—C11	61.74 (15)
C6^i^—C4—C5—O2	0.7 (3)	N1—C7—C12—C11	−177.88 (11)
C3—C4—C5—N1	1.92 (16)	C8—C7—C12—C11	−58.57 (15)

Symmetry codes: (i) −*x*, −*y*+1, −*z*+1.

### Hydrogen-bond geometry (Å, °)

**Table:**

*D*—H···*A*	*D*—H	H···*A*	*D*···*A*	*D*—H···*A*
C14—H12···O2^ii^	0.95	2.52	3.4349 (19)	161

Symmetry codes: (ii) *x*, −*y*+3/2, *z*−1/2.
